# EXAMINING COMMUNITY-BASED SERVICES DISCONNECTS IN LATE OLD AGE: PATHS FOR REACH THROUGH THE COMMUNICATION ECOLOGY

**DOI:** 10.1093/geroni/igz038.3302

**Published:** 2019-11-08

**Authors:** Carrie Leach, Julie Novak

**Affiliations:** 1 Institute of Gerontology, Wayne State University, Detroit, Michigan, United States; 2 Wayne State University, Detroit, Michigan, United States

## Abstract

As potentially eligible recipients continue to increase in number, understanding service system utilization and barriers can help ensure very old adults can access support from their communities when needed. A communication disjuncture between seniors and community-based service providers was revealed through a multi-year, county-wide older adult needs assessment utilizing data from 1,870 individuals. In response, officials convened a post assessment team that formed the three-person Community Advisory Group (CAG, all ≥69 years) who participated in this community-based participatory research (CBPR) study. This applied, qualitative study, guided by an ecological health communication research framework, conducted multilevel examinations of interactions among older adults and their social environment. Twenty in-depth, face-to-face, semi-structured interviews (mean = 82.5 years) were conducted based on a critical threshold of understanding achieved via researcher immersion in the community preceding this study, data collected, and CAG insight revealed through collaborative analysis. Communication Infrastructure Theory helped to reveal how participants’ diminishing social network interrelated with the communication environment acted to impede connections to services. In addition to utilization impediments, enabling elements of the communication infrastructure were identified so those resources might be leveraged to bridge the senior-provider divide. Findings from this study suggest new outreach approaches for connecting to older adults through their communication ecology. The findings add to the growing convergence of evidence that calls for improved communication with older adults to minimize poor interactions that hinder accessing resources that may enhance their social, emotional, and physical well-being.

